# Bst DNA Polymerase: Structure, Properties and Engineering Strategies in LAMP

**DOI:** 10.3390/ijms27104261

**Published:** 2026-05-11

**Authors:** Ekaterina Tikhonova, Anna Popinako, Aleksey Sazonov

**Affiliations:** Scientific Center of Genetics and Life Sciences, Sirius University of Science and Technology, 354340 Sirius, Russia; et@bio-dive.com (E.T.); sazonov.ae@talantiuspeh.ru (A.S.)

**Keywords:** Bst DNA polymerase, structure, molecular biology, thermal stability, DNA amplification

## Abstract

Bst DNA polymerase is a biotechnologically modified thermostable enzyme from the thermophilic Gram-positive bacterium *Geobacillus stearothermophilus*. The unique structure of Bst DNA polymerase determines its thermal stability, ability to replace a DNA strand and specificity. The high specificity of Bst DNA polymerase ensures the efficiency, sensitivity, and high rate of loop-mediated isothermal amplification (LAMP), which is widely used in vitro biotechnology. The review reveals the structural and functional features of the enzyme, its application in LAMP and methods of improvement of thermal stability (including directed evolution, site-directed mutagenesis, fusion constructs, and chemical modifications). The terminal transferase activity and ab initio synthesis are discussed regarding problems of Bst DNA polymerase and the ways to eliminate them. The questions of introducing modified nucleotides and primers to expand the diagnostic capabilities of LAMP are also discussed. Modern advances in Bst DNA polymerase engineering pave the way for the creation of reliable, thermostable, and highly specific test systems suitable for widespread diagnostic applications.

## 1. Introduction

DNA polymerases are enzymes that catalyze the synthesis of DNA molecules from deoxyribonucleotides using a DNA template. They play an important role in the preservation and transmission of the genetic information [1]. The participation of DNA polymerases in DNA replication, recombination, and repair determines the fundamental characteristics of all living things and ensures the process of evolution on Earth. Owing to their fundamental importance, DNA polymerases have been extensively studied, and numerous comprehensive reviews have described their structure, function, and biological roles [2,3]. In addition to their biological significance, DNA polymerases are key tools in modern biotechnology.

The history of DNA polymerase research is complicated and involves contributions from many laboratories. A major advance was achieved in 1956 when Arthur Kornberg and his colleagues isolated the first DNA polymerase from *Escherichia coli*, later named DNA polymerase I [4]. For their discoveries concerning the mechanisms of biological synthesis of ribonucleic and deoxyribonucleic acids, Arthur Kornberg and Severo Ochoa were awarded the Nobel Prize in Physiology or Medicine in 1959 [5]. While the work of the Kornberg laboratory was foundational, numerous other researchers made critical contributions to elucidating the mechanisms of DNA replication. Subsequent studies by many laboratories led to the identification and classification of DNA polymerases into several families based on sequence and structural similarities. To date, seven major families have been described: A, B, C, D, X, Y, and reverse transcriptases (RTs) [6]. Of these, enzymes based on DNA polymerases from families A and B are actively used in molecular biology (polymerase chain reaction, sequencing, molecular cloning, DNA synthesis, etc.) [7].

**Figure 1:**
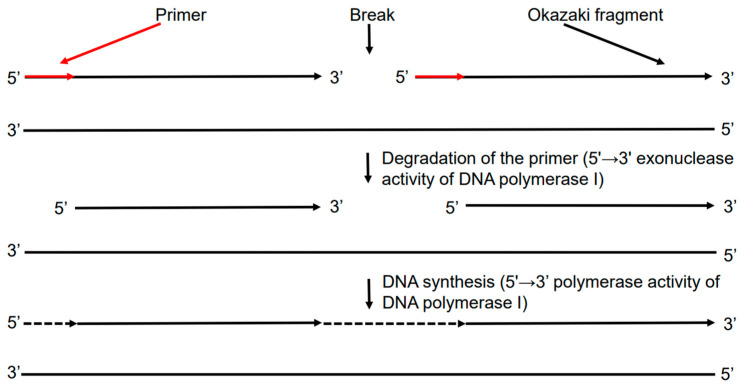
Nick translation scheme.

The widespread group of DNA polymerases among prokaryotes is DNA polymerase I, family A [8]. In bacteria, DNA polymerase I plays a role in repair, but its central and unique function is to complete the replication of the lagging strand of DNA. This process, known as “nick translation” (Figure 1), is possible due to the ability of DNA polymerase I to combine two enzymatic activities in a single reaction. By interacting with the nick at the junction of the RNA primer and the Okazaki fragment, the enzyme initiates conjugate hydrolysis and synthesis reactions. On the one hand, its 5′→3′ exonuclease activity degrades the RNA primer, creating a single-stranded region on the template; on the other hand, 5′→3′ polymerase activity immediately uses this freed-up template for the DNA synthesis. Thus, DNA polymerase I does not perform substitution, but rather replacement of ribonucleotides with deoxyribonucleotides, which makes the process of replication completion energy-efficient and minimizes the risk of dangerous intermediate structures forming [9]. Importantly, although DNA polymerase I provided key insights into DNA synthesis, it is not the primary enzyme responsible for chromosomal DNA replication in bacteria. Instead, the main replicative enzyme is the DNA polymerase III holoenzyme, family C, which carries out the bulk of DNA synthesis during bacterial genome duplication [10].

The importance of DNA polymerases in biotechnology was further highlighted by the development of the polymerase chain reaction (PCR), for which Kary B. Mullis was awarded the Nobel Prize in Chemistry in 1993 [11]. A key advancement that enabled PCR to become a universal laboratory technique was the introduction of the thermostable Taq DNA polymerase from the thermophilic bacterium *Thermus aquaticus* [12]. Taq DNA polymerase has been widely used to create multiple copies of a specific DNA fragment (amplification) during PCR, thanks to its resistance to high temperatures since then. Taq DNA polymerase catalyzes DNA synthesis on a single-stranded DNA template. Thus, the start of DNA synthesis in PCR is only possible after a preliminary stage of denaturation of double-stranded DNA at high temperature. The cyclic temperature changing in PCR requires considerable time and energy. Hence, PCR is difficult to use outside the laboratory.

The discovery of a new thermostable enzyme, Bst DNA polymerase, has led to the development of a new method of isothermal nucleic acid amplification: LAMP [13].

Bst DNA polymerase is a thermostable enzyme originally obtained from the thermophilic Gram-positive bacterium *Bacillus stearothermophilus* (currently *Geobacillus stearothermophilus*). In its native form, DNA polymerase I contains an N-terminal 5′→3′ exonuclease domain that participates in nick translation during DNA repair. However, in the biotechnologically engineered Bst DNA polymerase used in molecular biology, this exonuclease domain has been removed. A similar strategy was first demonstrated with *E. coli* DNA polymerase I, where proteolytic cleavage of the N-terminal exonuclease domain produced the Klenow fragment.

In contrast, the 5′→3′ exonuclease activity of Taq DNA polymerase is compatible with PCR conditions, as cyclic thermal denaturation enables repeated rounds of primer annealing and extension. In LAMP, however, this activity is undesirable because the reaction proceeds at a constant temperature and relies on stable stem-loop structures and continuous strand displacement. Degradation of primers or looped DNA intermediates disrupts the amplification process and reduces overall efficiency.

As the result of this domain deletion, Bst DNA polymerase lacks 5′→3′ exonuclease activity and exhibits strong strand displacement activity, meaning it can synthesize DNA while simultaneously displacing the downstream DNA strand. Structural studies of DNA polymerases with intrinsic strand displacement activity suggest that a flexible enzyme architecture enables continuous DNA synthesis by locally destabilizing downstream duplex DNA at the single- to double-stranded junction during translocation [14], while for Bst DNA polymerase, such detailed structural characterization remains more limited.

Bst DNA polymerase is characterized by 10 times higher processivity compared to Taq DNA polymerase, contributing to rapid and efficient DNA synthesis [15]. Due to its simplicity, speed, sensitivity, and accuracy, the LAMP method is one of the most promising tools for molecular diagnostics and has a wide range of applications in science, medicine, veterinary medicine, industry (agriculture, food industry), criminalistics, environmental monitoring, and other areas of biotechnology [16,17].

**Figure 2:**
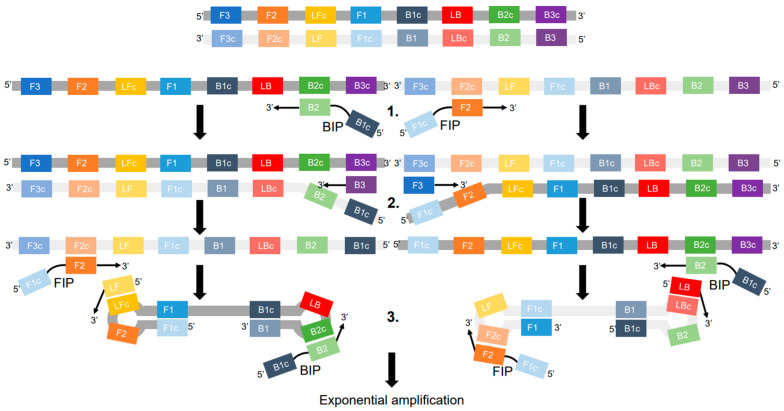
Schematic representation of the LAMP mechanism. 1. The inner primers (FIP and BIP) first bind to the target DNA and initiate strand synthesis via Bst DNA polymerase. 2. The outer primers (F3 and B3) displace the newly synthesized strands, generating single-stranded DNA fragments. 3. The inner primers (FIP and BIP) then bind to the products from the previous step and initiate further strand synthesis. Due to the complementarity of sequences at the ends of these fragments, the molecules self-anneal to form characteristic “dumbbell” structures. These structures serve as templates for inner and loop primers (LF and LB), enabling rapid cyclic DNA synthesis and resulting in exponential amplification and accumulation of amplicons of various lengths.

The LAMP method was described in 2000 by Japanese scientists led by Tsugunori Notomi [13]. The essence of this method (Figure 2) is to perform analysis at a constant temperature of 60–65 °C using a set of six primers targeting eight regions (F3, R3, FIP, BIP, LF, LB). At the initial stage, a dumbbell-shaped DNA structure is formed, which serves as a template for exponential DNA amplification. In this case, the reaction product is a set of concatemers of different lengths. Due to enhanced resistance of Bst DNA polymerase to a broad range of substances that typically inhibit PCR, LAMP can be performed without the laborious and time-consuming stage of DNA/RNA isolation. The isothermal reaction does not require expensive equipment, and the signal can be detected using intercalating dyes, [18] or turbidimetrically [19] by the formation of a precipitate. Despite its advantages, LAMP has a number of practical problems, which we discuss in detail in this review.

This review provides an integrated overview of the structural and functional properties of Bst DNA polymerase. Particular emphasis is placed on its intrinsic terminal transferase and ab initio activities, as well as on modern enzyme engineering strategies and the use of modified primers and nucleotides to improve the specificity and diagnostic performance of LAMP-based assays.

## 2. Structure of Bst DNA Polymerase

**Figure 3:**
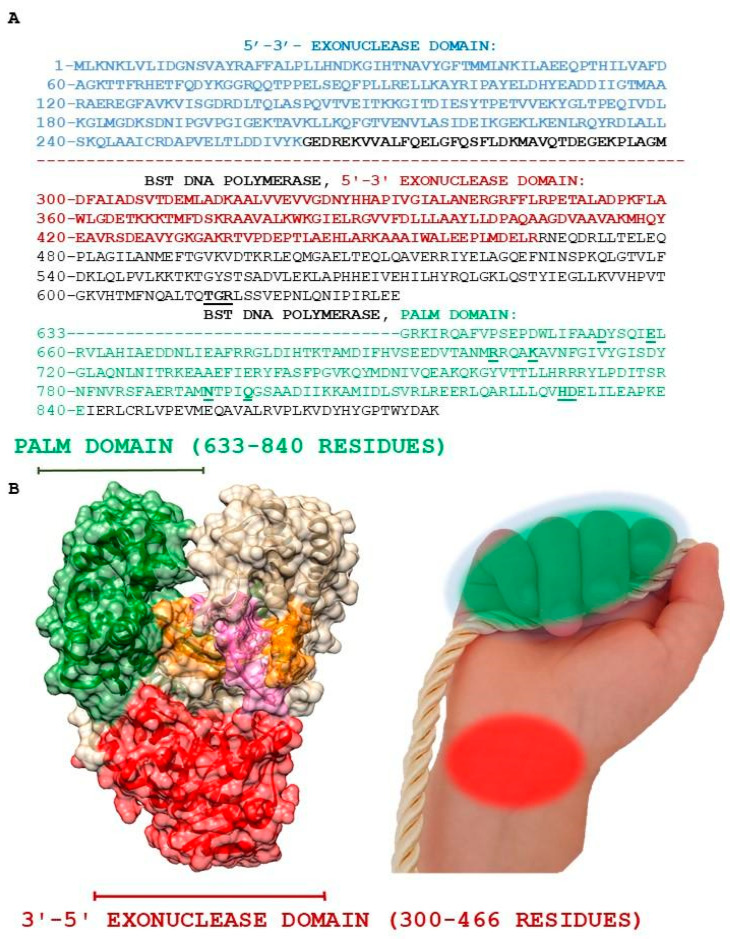
Amino acid sequence of DNA polymerase I from *G. stearothermophilus* (**A**) and the structure of Bst DNA polymerase (PDB ID 6DSU) domains (**B**) are highlighted according to the InterPro classification https://www.ebi.ac.uk/interpro/ (accessed on 25 October 2025). 5′-3′ exonuclease domain (blue); 3′-5′ exonuclease domain (red); palm domain (green). Conserved residues among family A of DNA polymerases [8] are underlined (**A**); domain organization of Bst DNA polymerase (PDB ID 6DSU) visualized using UCSF Chimera (**B**). The primer is shown in orange. Beige-colored residues correspond to fragments forming the structural thumb and palm subdomains, which are also indicated schematically; the photo of the fist was created by the authors (**B**).

In 1972, the first paper on DNA polymerase I from *G. stearothermophilus* was published [20]. Later, in 1987, S. Y. Ye and G. F. Hong performed proteolysis of DNA polymerase I with subtilisin in the search for a more thermostable protein to improve DNA sequencing results. The enzyme was divided into two fragments: an N-terminal domain with 5′-3′ exonuclease activity and a C-terminal fragment called the “large fragment” (Bst Fragment, BF, 587 amino acid residues) (Figure 3) [21]. The BF fragment obtained from *G. stearothermophilus* was later named Bst DNA polymerase. Due to its higher activity at 65 °C compared to the original DNA polymerase I, Bst DNA polymerase has been used in LAMP since 2000 [13].

In 1997, X-ray crystallography was used to determine the structure of Bst DNA polymerase with a resolution of 2.1 Å. It corresponds to high resolution and allows the three-dimensional structure of the protein to be reliably determined. James Kiefer and co-authors [15] identified two structural domains: the 3′-5′ exonuclease and polymerase domains, which are characteristic of all members of the DNA polymerase I family. Despite sequence variability, all currently known DNA polymerases I have a similar architecture resembling a right hand. The “palm” subdomain contains the active center of the polymerase. The “fingers” subdomain participates in the binding of the template and/or deoxynucleotide triphosphates (dNTPs), while the “thumb” subdomain ensures the high processivity of Bst DNA polymerase by restricting the movement of DNA between the active sites of the polymerase and exonuclease (Figure 3) [22]. The fragments corresponding to the “palm”, “fingers” and “thumb” subdomains are indicated schematically (Figure 3B). Historically, the names of these fragments are preserved solely for understanding the architecture of Bst DNA polymerase. Subdomains “fingers” and “thumb” are not represented in current InterPro classification. In this review, the structure of Bst DNA polymerase is based on the current InterPro classification (Figure 3): 3′-5′ exonuclease domain (300–466 residues); palm domain (633–840 residues).

**Figure 4:**
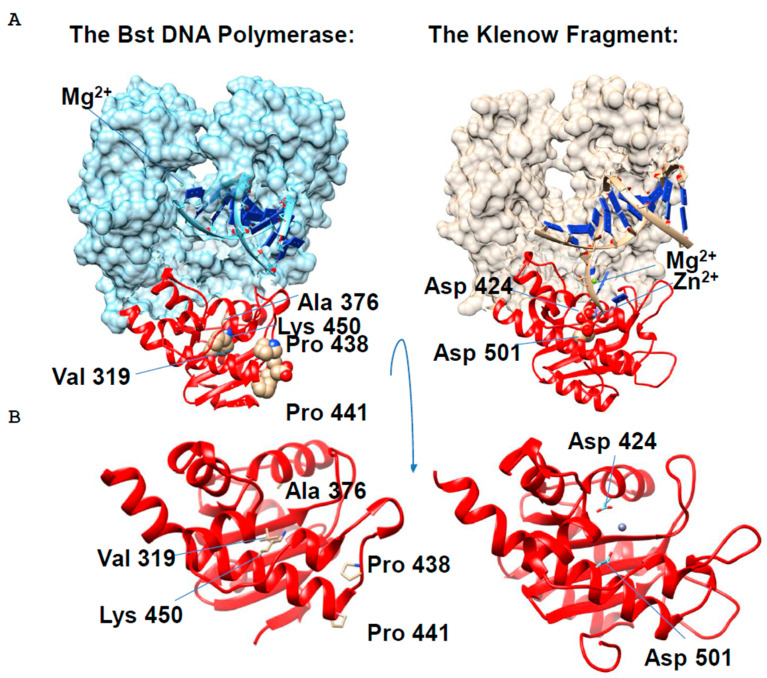
Structural features of the Bst DNA polymerase (PDBID 6DSU) in comparison with the Klenow fragment (PDBID 1KLN, 8OO6) (**A**), zooming in on the 3′-5′ exonuclease domain (**B**). The 3′-5′ exonuclease domain is highlighted in red according to the InterPro domain classification. The loop Pro438-Pro441 of the Bst DNA polymerase deforms the exonucleases cavity, while Val319, Ala376, and Lys450 residues do not bind magnesium ions, and the result is the absence of 3′-5′ exonuclease activity. The residues Asp 424 and Asp 501 of the Klenow fragment bind divalent ions.

Bst DNA polymerase does not possess 3′-5′-exonuclease activity [13]. Through a comparative structural analysis, James Kiefer and his co-authors identified the amino acid residues in the N-terminal 3′-5′ exonuclease domain of Bst DNA polymerase that cause it to lack an editing function. They then contrasted this with the structure of the active 3′-5′ exonuclease domain of the Klenow fragment (Figure 4).

The comparison revealed key differences in the structure of the active centers of the 3′-5′ exonuclease domain of the two enzymes. In Bst DNA polymerase, residues Val319, Ala376, and Lys450, located in a position corresponding to the active center, do not carry a negative charge (Figure 4). This prevents them from coordinating magnesium cations (Mg^2+^), which are necessary cofactors for the hydrolysis of DNA phosphodiester bonds. An additional factor hindering catalysis is the deformation of the exonuclease cleft due to the rotation of the Pro438-Pro441 loop.

In contrast, the active site of the 3′-5′ exonuclease domain of the Klenow fragment contains negatively charged aspartic acid residues (Asp424 and Asp501) that effectively bind Mg^2+^ ions (Figure 4). Analysis has shown that this particular organization of the metal-binding center is critical for the manifestation of 3′-5′-exonuclease activity [23].

The absence of 3′-5′ exonuclease activity in Bst DNA polymerase was also considered in terms of the adaptation of *G. stearothermophilus* to high-temperature conditions. F. K. Loier and co-authors compared the characteristics of DNA polymerase I molecules from mesophilic (*E. coli*), thermophilic (*G. stearothermophilus*) and hyperthermophilic (*T. aquaticus*) organisms. The following patterns were discovered: the molecular weight of DNA polymerases I decreases with an increase in the temperature optimum. Deletions in DNA polymerase I from *G. stearothermophilus* (52 amino acid residues) and Taq DNA polymerase (96 amino acid residues) are located in the 3′-5′ exonuclease domain [24] and lead to a loss of its activity.

Loyer F.S. and co-authors suggested that the reduction of the 3′-5′ exonuclease domain sequence in thermophilic and hyperthermophilic organisms and the loss of its activity are associated with the adaptation of organisms to high temperatures. DNA polymerases I “give” the exonuclease activity in order to gain thermal stability [25]. The research of F. K. Loier and co-authors was based on a comparison of three structures. Thus, the modern phylogenetic analysis is essential to confirm Loyer F. S.’s suggestion.

However, the functional significance of the 3′-5′ exonuclease domain is not limited to evolutionary compromise. The 3′-5′ exonuclease domain of Klenow DNA polymerase I is critical for maintaining catalytic efficiency. Complete removal of the 3′-5′ exonuclease domain led to a 10-fold decrease in the catalytic activity of Klenow DNA polymerase, indicating its contribution to polymerase processivity [26,27]. The role of the 3′-5′ exonuclease domain in the catalytic activity of Bst DNA polymerase needs to be studied.

**Figure 5:**
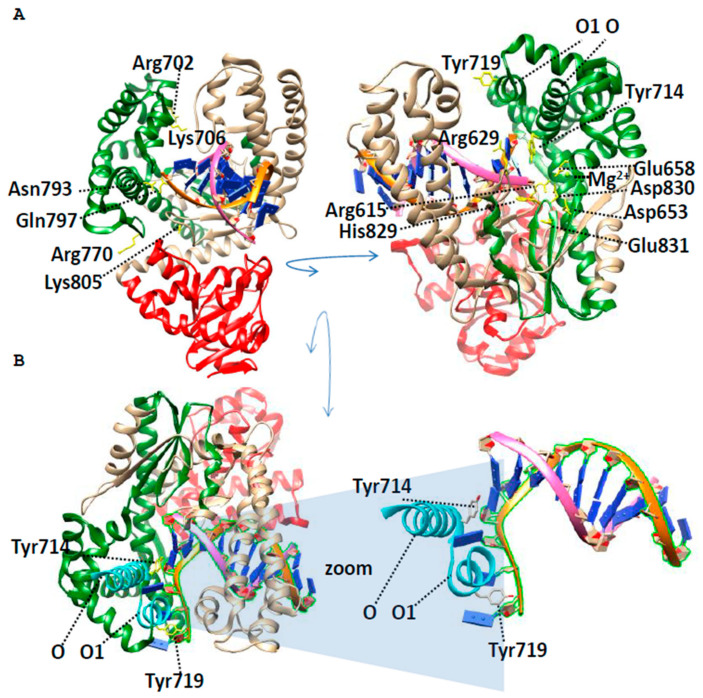
The pre-insertion complex structure of DNA polymerase I from *G. stearothermophilus* at ((**A**), PDB ID 6DSU) and the time-resolved structure of Bst DNA polymerase, 4 hr post dATP and dCTP addition ((**B**), PDB ID 7K5S), where domains are highlighted according to the InterPro classification https://www.ebi.ac.uk/interpro/ (accessed on 25 October 2025): 3′-5′ exonuclease domain (red); palm domain (green). Conserved residues among family A of DNA polymerases [8] are highlighted with yellow; domain organization of Bst DNA polymerase visualized using UCSF Chimera. The primer is shown in orange. Structural features of Bst DNA polymerase and functional roles of specific residues are highlighted: the substitution of Lys431 increases enzyme thermostability [28], and the residue Arg629 interacts with modified dNTPs [29], while Tyr714 and Tyr719 participate in DNA coordination [1], and the O(698–714)–O1(717–726) loop (cyan) acts as a pawl, zooming in on the O-O1 loop (**B**) [30].

By April 2026, a total of 56 structures of Bst DNA polymerase *G. stearothermophilus* and its mutants had been determined and published in the PDB database (PDB ID 1L3S, 1LV5, 1U45, 2BDP, 2HHQ, 2HHW, 2HVH, 2XO7, 2XY5, 2XY6, 2XY7, 2Y1I, 3EYZ, 3HP6, 3HPO, 3HT3, 4B9L, 4DSI, 4DSJ, 4O0I, 4UQG, 4YFU, 6DSV, 6DSW, 6DSX, 6DSY, 6DSU, 6MU4, 6MU5, 6P5C, 6UEU, 6UR2, 6UR4, 6UR9, 6US5, 7K5O, 7K5P, 7K5Q, 7K5R, 7K5S, 7K5T, 7K5U, 8SCG, 8SCI, 8SCJ, 8SCK, 8SCL, 8SCM, 8SCN, 8SCO, 8SCP, 8SCQ, 8SCR, 8SCS, 8SCT, 8SCU). However, the molecular mechanism of Bst DNA polymerase has not yet been fully elucidated. The directed mutagenesis allows conclusions to be drawn only about the role of individual residues (Figure 5).

The detailed description of the structure of Bst DNA polymerase was represented in the work of Kiefer with co-authors [15]. Conserved residues coordinating Mg^2+^ ions necessary for the catalysis of phosphodiester bond formation (Asp653, Asp830, Glu831, Figure 5) were described here for the first time.

The next work of Kiefer with co-authors [31] presents high-resolution crystal structures of Bst DNA polymerase with DNA primer templates bound productively at the polymerase active site and partly reveals the mechanism of high-fidelity DNA replication. These structures allow us to describe all interactions between DNA and the active site of Bst DNA polymerase.

The structures of complexes of Bst DNA polymerase with different substrates allow us to reveal about five different active sites [32,33], and basic and intermediate conformational states on the DNA synthesis pathway [34].

The time-resolved X-ray crystallography revealed the reaction pathway of a replicative DNA polymerase to elucidate the order and transition between intermediates [30]. The translocation step appears to follow a push–pull mechanism where the O(698–714)–O1(717–726) loop (Figure 5) acts as a pawl to facilitate unidirectional movement along the template with conserved tyrosine residues 714 and 719 functioning as tandem gatekeepers of DNA synthesis. Thus, the conserved tyrosines realize the widespread mechanism in the work of the enzymes: which connect two enzyme subdomains into an active conformation [35].

The other conserved residues of the Palm domain Arg702, Lys706, Arg770, Gln797, Asn793, and Lys805 interact with the DNA strand and cut out erroneous pairs during synthesis, as well as form a hydrophilic environment for the substrate [8]. Conservative residues of the Palm domain, Asp653, Asp830, and Glu831, which coordinate Mg^2+^ ions, are necessary for the catalysis of phosphodiester bond formation. The residues Arg615, Arg629, Glu658, and His829, which bind DNA and stabilize the structure of the active site, are also conserved [36].

The structural basis of the molecular mechanisms of Bst DNA polymerase function is more complex than a combination of residues in the active site. The coordinated work of the Bst DNA polymerase and its environment is a complex mechanistic puzzle that remains to be solved in the future.

## 3. Properties of Bst DNA Polymerase

**Figure 6:**
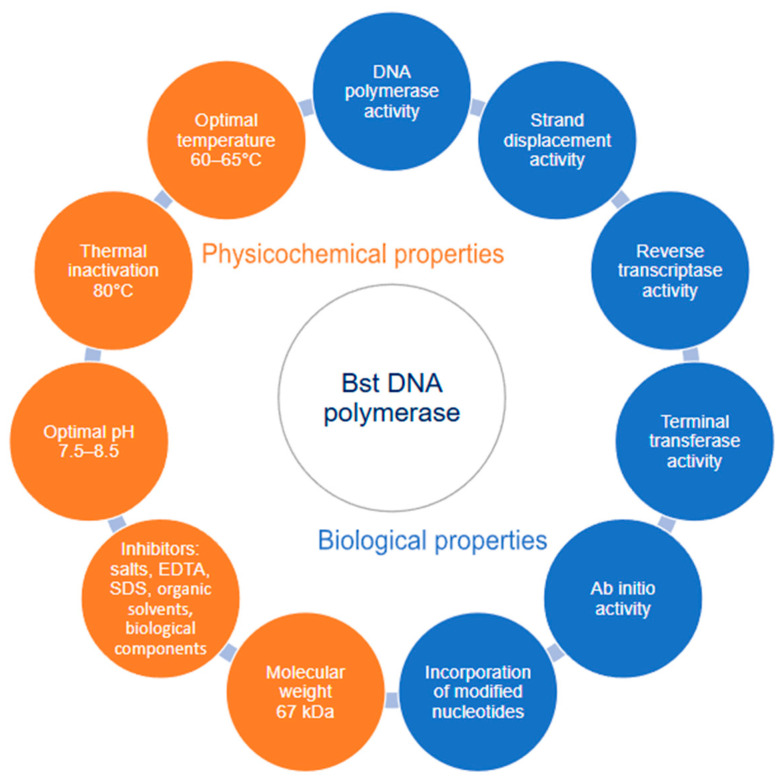
Circular schematic summarizing the key physicochemical and biological properties of Bst DNA polymerase. Created by the author using Microsoft PowerPoint Online (Microsoft Corporation, Redmond, Washington, DC, USA).

The key physicochemical and biological properties of Bst DNA polymerase that determine its widespread application in isothermal amplification methods are summarized in Figure 6.

Bst DNA polymerase is a protein with a molecular weight of 67 kDa. The enzyme exhibits the maximum of the polymerase activity in the temperature range from 60 to 65 °C [21]. Conducting the reaction at temperatures below 55 °C leads to a significant decrease in the synthesis rate and a sharp increase in non-specific products due to the weakening of primer annealing stringency. Bst DNA polymerase demonstrates high thermal stability due to its origin from a thermophilic microorganism. Complete and irreversible inactivation of the enzyme is observed during prolonged incubation (more than 15 min) at temperatures exceeding 80 °C. However, a progressive decrease in enzymatic activity is already observed at temperatures above 70 °C. The activity of Bst DNA polymerase significantly depends on the acidity of the reaction medium. The maximum of catalytic efficiency is observed in the pH range of 7.5–8.5 [37]. Standard Tris-HCl-based reaction buffers stabilize the pH in this range. Bst DNA polymerase inhibitors include chelators of divalent cations, such as EDTA and EGTA. They effectively bind ions that act as cofactors. High concentrations of salts, for example, (NH_4_)_2_SO_4_ > 50 mM, NaCl > 100 mM, have an inhibitory effect by disrupting the ionic and hydrophobic interactions that stabilize the enzyme structure. Some detergents, such as SDS, cause complete inactivation at low concentrations due to the protein denaturation. In addition, Bst DNA polymerase is also susceptible to organic solvents and components derived from biological samples, such as heme from blood, humic acids from soil, polysaccharides, and endogenous inhibitor proteins present in tissue extracts [38]. Data on the main biochemical characteristics have been reported [15]. Its specific activity is 1.5  ×  10^5^ U/mg on Calf thymus DNA and 4.9  ×  10^5^ U/mg on Primed M13, Kcat is 191.2 (39.3) total dNTP/sec, Km DNA is 3.4 (1.6) nM, Km dNTP is 13 (5.5) μM and processivity is 111 nt.

Magnesium, cobalt, manganese, and cadmium ions can act as cofactors for Bst DNA polymerase. The error rate when using magnesium ions as a cofactor is the lowest compared to other cofactors. Manganese ions reduce amplification efficiency and increase the error rate, but allow Bst DNA polymerase to exhibit more effective reverse transcriptase activity [39].

DNA polymerases I of the A family, along with their main DNA-dependent DNA polymerase activity, are characterized by their ability to use RNA as a template. As mentioned earlier, the reverse transcriptase activity of Bst DNA polymerase can be significantly increased in the presence of Mn^2+^ ions. However, it has been found that during cDNA synthesis of more than 65 bp, the efficiency of reverse transcription using Bst DNA polymerase is significantly reduced compared to Avian Myeloblastosis Virus Reverse Transcriptase (AMV RT) [40].

Strand displacement activity is the ability of DNA polymerase to dissociate DNA duplexes and displace the leading strand during synthesis. It ensures continuous elongation without the need for preliminary denaturation of the duplex by external factors, such as heating, or helicases. The high strand displacement activity of Bst DNA polymerase is a key property that determines its widespread use in LAMP. The structural basis for this activity in Bst DNA polymerase remains unknown: to date, there are no studies describing in detail the molecular mechanism of strand displacement by this enzyme [1].

Bst DNA polymerase, like some other group A DNA polymerases, has terminal transferase activity. This is an activity in which nucleotides can be incorporated into the nascent DNA chain independently of the template [41]. In this case, dATP is predominantly incorporated [42]. For example, in the case of Taq DNA polymerase, this activity allows PCR products to be cloned without prior treatment with restriction enzymes, or exonucleases. However, in the case of Bst DNA polymerase, terminal transferase activity is a negative factor in LAMP, causing the formation of non-specific amplification products.

Bst DNA polymerase also possesses ab initio activity [43]. This activity is found in thermophilic DNA polymerases and leads to the formation of DNA from free dNTPs in the absence of a template and primers. It is believed that ab initio activity appeared early in evolutionary development [44]. In 2004, Singo Liang, Kari Jensen and Maxim D. Frank-Kamenetsky showed that restriction endonucleases significantly increase the ab initio activity of Bst DNA polymerase [45]. The presence of DNase I, nucase, or DnaB helicase also stimulated ab initio synthesis [46]. For this reason, it is extremely important to pay special attention to the purification of recombinant Bst DNA polymerase from endogenous *E. coli* enzymes. However, even in the absence of additional enzymes, ab initio synthesis leads to false positive results and reduces the sensitivity of LAMP-based test systems.

Bst DNA polymerase has a property that allows it to expand its range of applications. Along with natural dNTPs, Bst DNA polymerase is capable of incorporating modified nucleoside triphosphates. It has been established that modified dNTPs with π-electron substituents (vinyl, ethynyl, phenyl) have a greater affinity for the active site of Bst DNA polymerase than natural dNTPs, due to anion-π interaction with the amino acid Arg629 (Figure 5) [29]. Bst DNA polymerase is also capable of incorporating 2′-deoxyadenosine-5′-(α-thio)-triphosphate (dATPaS) [47] and 2′-deoxynucleoside-5′-(α-P-seleno)-triphosphate (dATPaSe). In the study by F. Ekstein and J. B. Thomson, it was reported that most DNA and RNA polymerases use the Sp isomer of dATPaS exclusively [48]. The same pattern is observed in the case of dATPaSe: Bst DNA polymerase effectively recognizes the Sp isomer of dATPaSe, while the Rp isomer cannot be used as a substrate but does not inhibit the reaction. Hence, there is no need to perform stereoselective separation of diastereomers [49,50].

## 4. Biotechnological Production and Practical Applications of Bst DNA Polymerase in LAMP

Although LAMP is widely used in molecular diagnostics, its performance is still limited by several biochemical factors. To provide a structured framework for the discussion below, Table 1 summarizes the main limitations of LAMP and the corresponding strategies for optimizing reported in the literature.

Bst DNA polymerase has found extensive application in LAMP. This method is widely used for the diagnosis of infectious diseases, in veterinary medicine, food safety control, and environmental monitoring. Typically, LAMP assays employ recombinant Bst DNA polymerase produced by an *E. coli* host strain. Currently, a wide range of commercial preparations of this enzyme are available on the market. At the same time, numerous studies have been devoted to optimizing the conditions for recombinant Bst DNA polymerase biosynthesis, with the aim of increasing yield and reducing production costs.

The authors of study [51] managed to produce up to 20% of Bst DNA polymerase from the total cell mass in the *E. coli* expression system. The highest protein yield was obtained after 4 h of cultivation at 23 °C following induction with isopropyl-β-D-1-thiogalactopyranoside (IPTG). In LAMP reactions, the enzyme demonstrated activity comparable to that of the commercial Bst 2.0 polymerase (New England Biolabs, NEB).

In another study [52], silica gel was used as a low-cost alternative to Ni-NTA affinity chromatography to reduce purification expenses. For this purpose, a recombinant construct was engineered containing a positively charged silaffin tag R5 to facilitate the binding to silica particles, along with the fluorescent protein mCherry fused to Bst DNA polymerase via a flexible linker (GGGGSGGGGS), enabling monitoring of the protein during purification by fluorescence. In addition to its cost-effectiveness, the use of negatively charged silica gel as a sorbent reduced residual *E. coli* DNA contamination. However, the overall protein yield decreased—4.38 ± 0.21 mg/L of *E. coli* culture compared to 9.77 ± 0.09 mg/L obtained using nickel resin purification.

Apart from IPTG induction, expression of Bst DNA polymerase can also be regulated under a rhamnose promoter [53] or by autoinduction using lactose [54].

In conclusion, the optimization of recombinant Bst DNA polymerase production conditions is a critical task that directly affects production cost and, consequently, the final price of LAMP-based diagnostic test systems. Therefore, improvement of Bst DNA polymerase production methods is not only a scientific and technological challenge but also an economically significant objective.

Alongside DNA pathogen detection, reverse transcription LAMP enables RNA analysis without a separate reverse transcription step [73]. However, this approach requires either a thermostable reverse transcriptase capable of functioning at 60–65 °C—the optimal temperature range for LAMP, or a Bst DNA polymerase with high reverse transcriptase activity.

The authors of study [74] developed a fusion Bst DNA polymerase exhibiting enhanced reverse transcriptase activity and increased tolerance to inhibitors. The fusion enzyme contains a hydrophobic protein Hp47, a DNA-binding protein Sto7d, and Bst DNA polymerase (BF). It was shown that the presence of Hp47 significantly increases the affinity of the polymerase for RNA.

Unfortunately, there are not structures of the fusion Bst DNA polymerases in PDB, but investigations of hydrophobic protein Hp47 as part of villin [55] reveal the significant role of aromatic and hydrophobic residues as the hydrophobic core improvement of the molecule. Thus, conservative hydrophobic and aromatic residues of villin due to hard core structuring take part in functional blocking of the free end of the actin filament. So, the presence of the Hp47 protein fragment adds the aromatic and hydrophobic residues in the total core of the structure of Bst DNA polymerase-fusion, which can probably increase tolerance to inhibitors and stabilize the molecule.

Bst DNA polymerase is also capable of utilizing modified oligonucleotides in LAMP reactions, thereby expanding the range of possible amplification conditions. For instance, the use of phosphorothioate oligonucleotides in combination with urea and single-strand binding proteins (SSBs) enabled LAMP to be performed at 40 °C with sensitivity and specificity comparable to those achieved at 65 °C [66].

Another promising application of Bst DNA polymerase is mutation detection. The PNA-LNA-LAMP technology integrates peptide nucleic acids (PNAs) and locked nucleic acids (LNAs). PNAs are synthetic DNA analogs in which the natural phosphodiester backbone is replaced with a non-hydrolyzable 2-aminoethylglycine chain. LNAs, on the other hand, are modified ribonucleotides characterized by a methylene bridge between the 2′-oxygen and 4′-carbon atoms of the ribose ring, conferring conformational rigidity and increased thermodynamic stability of the resulting duplexes [75].

PNA probes demonstrate high sensitivity to single-nucleotide mismatches due to the higher melting temperature (Tm) of fully complementary PNA–DNA duplexes compared to DNA–DNA duplexes [76]. Moreover, PNA molecules can be designed to selectively inhibit amplification of strictly complementary DNA templates. PNA-mediated LAMP has been successfully employed for detecting macrolide-resistant *Treponema pallidum* harboring mutations in the 23S rRNA gene [56]. The high hybridization specificity and structural stability of these molecules have enabled efficient molecular diagnostics of mutations, including detection of *KRAS* proto-oncogene variants in colorectal [57] and pancreatic cancers [58,59], as well as CALR type 1 and 2 mutations in patients with myeloproliferative neoplasms [60]. Nancy Sharma and colleagues further applied PNA-LNA-LAMP for identifying SNPs associated with fungicide QoI resistance in *Erysiphe necator* [61]. Thus, the use of Bst DNA polymerase in LAMP reactions with modified primers represents a promising direction for diagnostic applications.

Despite the high specificity and sensitivity of LAMP-based test systems, the generation of nonspecific amplification products, sometimes even in the absence of template DNA, remains a significant issue. Several causes of nonspecific product formation have been identified.

First, as noted previously, Bst DNA polymerase exhibits terminal transferase activity, enabling the addition of one or more deoxyribonucleotides to the 3′-end of a growing DNA strand in a template-independent manner. The resulting sequences may randomly form complementarity with other DNA regions in the reaction mixture, act as nonspecific primers, and initiate unintended synthesis. Consequently, a variety of artifact products differing in length and sequence may accumulate, competing with the target amplicons for primers, nucleotides, and enzyme molecules. Currently, the primary approach to addressing this issue involves empirical optimization of the reaction buffer composition; however, this strategy only partially suppresses artifacts and does not completely eliminate nonspecific amplification [62].

Second, Bst DNA polymerase exhibits ab initio activity [43]. Such spontaneous synthesis occurs not only at the enzyme’s optimal reaction temperature but also at lower temperatures, particularly during prolonged storage of the polymerase in the reaction mixture [77]. Researchers have found that using selenium-modified deoxynucleoside triphosphates (dNTPαSe) can inhibit ab initio synthesis and improve system sensitivity. This effect is attributed to the lower binding affinity of dNTPαSe for Bst DNA polymerase compared to natural dNTPs [63,64]. However, due to their high cost, this approach is not suitable for large-scale LAMP test production. Therefore, the search for more affordable analogs of dNTPαSe remains an important goal in optimizing LAMP reactions.

Finally, a major source of nonspecific products is the formation of primer dimers and hairpin structures: stable, double-stranded DNA regions formed through self-annealing or cross-annealing of primers. The number of such duplexes increases exponentially during LAMP reactions, leading to accumulation of byproducts. Because LAMP requires a greater number of primers than conventional PCR, careful primer design is particularly critical.

Inhibition of the enzyme’s active site can also significantly reduce nonspecific amplification. During reaction setup, primers may transiently and nonspecifically hybridize with each other, or with non-target sequences. If the polymerase is active under these conditions, synthesis begins immediately, amplifying these errors. Temporarily inhibiting the enzyme prevents DNA synthesis until reaction initiation. Researchers from China developed a light-controlled Bst DNA polymerase using an aptamer that blocks the enzyme’s active site; exposure to ultraviolet light cleaves the linker and activates the reaction [65].

In addition to careful primer design and active site inhibition, increasing the LAMP reaction temperature can further help to mitigate nonspecific amplification.

## 5. Engineering and Enhancement of Bst DNA Polymerases’ Thermostability

In 2023, Tyler L. Dangerfield and colleagues elucidated the kinetics of the main stages of the LAMP reaction. It was found that the rate-limiting step in LAMP initiation is primer annealing [78]. Consequently, higher reaction temperatures facilitate strand separation in double-stranded nucleic acids, promote more efficient primer-template hybridization, and accelerate reaction initiation. This approach enables faster diagnostic assays.

Elevated reaction temperatures also help reduce the formation of nonspecific secondary primer structures, thereby improving assay sensitivity. Moreover, the ability of the polymerase to tolerate short-term exposure to high temperatures creates opportunities for developing hot-start polymerases, allowing reaction setup at ambient temperature and reducing nonspecific product formation during storage and preparation [79]. For these reasons, enhancing the thermostability of Bst DNA polymerase represents a relevant and important biotechnological objective. Table 2 summarizes key strategies used to enhance the thermostability and functional performance of Bst DNA polymerase.

The first biotechnological approach to improving the thermostability of Bst DNA polymerase was reported by Pavlova A. R. et al. in 2012 [67]. The authors examined the effect of helix–hairpin–helix (HhH) domains on the thermostability of DNA polymerases, using variants of the C-terminal domain of topoisomerase V (Topo V) from *Methanopyrus kandleri* (hyperthermophile, with optimal growth at 98 °C). The addition of the C2 domain to the C-terminus of Bst DNA polymerase increased its half-life eightfold at temperatures up to 95 °C [67]. The C2 domain binds DNA via backbone interaction with the hairpin motif, improving DNA polymerase processivity, thermostability, and salt tolerance by strengthening the protein–DNA interaction. The HhH motif binds to DNA phosphate groups without sequence specificity of DNA, aiding protein interaction with the DNA backbone. The strong network of electrostatic interactions between charged residues of the C2 domain gives the system additional strength at high temperatures and in excess of salt.

In 2018, Milligan et al. [68] engineered two polymerase variants with strand-displacement activity using high-temperature isothermal compartmentalized self-replication (HTI-CSR). The first variant was a *T. aquaticus* DNA polymerase (Taq) lacking the 5′→3′ exonuclease domain and containing 13 amino acid substitutions derived from Bst DNA polymerase. The second variant was a Bst DNA polymerase carrying four amino acid substitutions. The first enzyme exhibited enhanced thermostability compared to native Bst DNA polymerase and withstood brief incubations at up to 92.5 °C but showed reduced LAMP efficiency. In contrast, the second variant demonstrated greater activity than the wild-type enzyme but was completely inactivated at 85 °C [68].

The combination of site-directed mutagenesis and chimeric protein design has proven effective in improving the properties of Bst DNA polymerase. For instance, introducing the K431E substitution and adding a DNA-binding domain (DBD) from *Pyrococcus abyssi* (hyperthermophilic archaeon with optimum growth temperature at 96 °C) to the N-terminus of Bst DNA polymerase increased the enzyme’s tolerance to LAMP inhibitors and its thermostability, while also doubling its catalytic activity [29]. The residue K431 probably binds the inhibitors (urea, NaCl, ethanol, heparin, EDTA) in wild-type Bst DNA polymerase, and the K431E substitution with the DNA-binding domain (DBD) from *Pyrococcus abyssi* stabilizes all structure of the mutagenic chimera at high temperatures.

Pike I. et al. developed a chimeric polymerase containing an actin-binding domain (HP47) from *Gallus gallus* at the N-terminus and three substitutions (S371D, T493N, A552G) in the Bst DNA polymerase sequence. These modifications enabled LAMP reactions to be conducted at 73 °C, substantially increasing reaction speed [69].

Chemical modification also represents an effective strategy for enhancing thermostability. For example, Bst DNA polymerase chemically modified with mPEG-ALD (methoxy-polyethylene-glycol-aldehyde) retained up to 80% activity at 70 °C and displayed increased tolerance to LAMP inhibitors such as urea, ethanol, sodium chloride, and potassium chloride. The enhanced stability of the enzyme under harsh conditions was attributed to conformational changes induced by PEGylation; however, the exact mechanism remains poorly understood [70].

Using fluorescence-activated droplet sorting (FADS), Li H. et al. achieved directed evolution of wild-type Bst DNA polymerase into more stable and functionally enhanced mutants. These variants exhibited increased thermostability, improved strand displacement activity, and accelerated LAMP kinetics—reducing the reaction time from 40 min to 10 min. The enzyme also remained stable in lyophilized form for up to two months at 50 °C and enabled high-temperature LAMP assays at 70 °C [71].

In 2025, a research group led by Simões R. S. R. M. conducted bioinformatic screening and gene synthesis to identify thermophilic homologs of Bst DNA polymerase. Phylogenetic analysis using BLASTP identified over 100 homologous sequences, from which 18 polymerases with >80% sequence identity, primarily from thermophilic bacteria, were selected for further study. The corresponding genes were cloned and expressed in *E. coli*. Experimental testing revealed three enzymes that retained activity at 72.5 °C, exhibited high inhibitor resistance, and reduced nonspecific product formation during LAMP [72].

Collectively, these studies demonstrate that improving the thermostability of Bst DNA polymerase directly enhances the efficiency, reliability, and speed of LAMP assays.

## 6. Perspectives

Bst DNA polymerase, the large fragment of *G. stearothermophilus* DNA polymerase I, occupies a unique position among modern molecular biotechnology tools. Its distinctive structural features, the absence of a 5′→3′ exonuclease domain and a nonfunctional 3′→5′ exonuclease domain, endow it with the key properties required for isothermal amplification: high processivity and strong strand displacement activity. These characteristics have established Bst DNA polymerase as an indispensable component of LAMP, conferring advantages in reaction speed, sensitivity, and suitability for field diagnostics.

However, the enzyme’s utility is limited by its intrinsic terminal transferase and ab initio activities, which contribute to nonspecific amplification products and reduce assay specificity. Another significant constraint is its moderate thermostability, which narrows the operational temperature range for LAMP and increases the likelihood of nonspecific synthesis during reaction setup.

Modern strategies in protein engineering, including directed evolution, site-directed mutagenesis, chimeric fusion with thermostable DNA-binding domains, and chemical modification, have yielded impressive results in enhancing the thermostability, specificity, and processivity of Bst DNA polymerase. Concurrently, the use of modified primers and nucleotides has enabled the creation of highly specific test systems for single-nucleotide polymorphism detection and has further optimized LAMP efficiency.

Future research should focus on an in-depth understanding of Bst DNA polymerase structure and catalytic mechanisms, forming the foundation for the rational design of improved enzyme variants. The development of polymerases with programmable properties will pave the way for next-generation high-precision diagnostic systems meeting the highest standards of analytical sensitivity, specificity, and operational robustness.

## Figures and Tables

**Table 1:** Summary of LAMP limitations and optimization strategies.

Limitation	Underlying Mechanism (Cause of Limitation)	Solution	Representative Study
Cost limitations	Complex expression and purification	Expression optimization; low cost purification	[51,52,53,54]
Limited RT efficiency	Weak RT activity	Fusion enzymes (Hp47, Sto7d)	[55]
Limited specificity of LAMP for SNP detection	Reduced mismatch discrimination of DNA–DNA hybridization under isothermal conditions	PNA-LNA-LAMP	[56,57,58,59,60,61]
Nonspecific amplification	Terminal transferase activity	Buffer optimization	[62]
	Ab initio DNA synthesis	Modified dNTPs (dNTPαSe)	[63,64]
	Self-/cross-hybridization of primers	Improved primer design; higher temperature	–
	Active enzyme at room temperature	Aptamer-controlled enzyme	[65]
Narrow temperature range	Significant decrease in the synthesis rate at temperatures below 55 °C	Phosphorothioate oligonucleotides + urea + SSB proteins	[66]
Slow reaction initiation	Primer annealing is rate-limiting	Thermostable variants of Bst DNA polymerase	[67,68,69,70,71,72]

**Table 2:** Strategies for improving thermostability of Bst DNA polymerase (↑ = increase, ↓ = decrease).

Approach	Strategy/Modification	Effect on Enzyme Properties	Representative Study
Domain fusion + site-directed mutagenesis	Fusion of C-terminal C2 domain from *Methanopyrus kandleri* Topo V	↑ thermostability (8× half-life at 95 °C), ↑ processivity, ↑ salt tolerance	[67]
	K431E substitution + DBD from *Pyrococcus abyssi*	↑ thermostability, ↑ inhibitor tolerance, ~2× catalytic activity	[29]
	HP47 domain (actin-binding) + S371D/T493N/A552G substitutions	Enables LAMP at 73 °C, ↑ reaction speed	[69]
Chemical modification (PEGylation)	mPEG-ALD conjugation	↑ thermal stability (80% activity at 70 °C), ↑ inhibitor resistance; mechanism partly unclear	[70]
Directed evolution	Mutagenesis and selection of Taq- and Bst-derived variants (HTI-CSR)	Altered thermostability; trade-off between stability and LAMP efficiency	[68]
	Fluorescence-activated droplet sorting of mutants	↑ thermostability, ↑ strand displacement, faster LAMP (40 → 10 min), long-term stability	[71]
Bioinformatic screening + genome mining	Identification and expression of thermophilic homologs	Discovery of enzymes active at 72.5 °C, ↑ inhibitor resistance, ↓ nonspecific amplification	[72]

## Data Availability

No new data were created or analyzed in this study. Data sharing is not applicable to this article.

## Abbreviations

The following abbreviations are used in this manuscript:

LAMP	Loop-mediated isothermal amplification
Kcat	Catalytic rate constant
Km	Michaelis constant
